# An Experimental Study on Fabricating an Inverted Mesa-Type Quartz Crystal Resonator Using a Cheap Wet Etching Process

**DOI:** 10.3390/s130912140

**Published:** 2013-09-10

**Authors:** Jinxing Liang, Jia Huang, Tian Zhang, Jing Zhang, Xuefeng Li, Toshitsugu Ueda

**Affiliations:** 1 Key Laboratory of Micro-Inertial Instrument and Advanced Navigation Technology, Ministry of Education, School of Instrument Science and Engineering, Southeast University, Nanjing 210096, China; E-Mails: hj18795899015@sina.cn (J.H.); 213102232@seu.edu.cn (T.Z.); 213101716@seu.edu.cn (J.Z.); 2 School of Electronics and Information Engineering, Tongji University, Shanghai 201804, China; E-Mail: lixuefeng81@hotmail.com; 3 Graduate School of Information, Production and System, Waseda University, Kitakyushu 808-0135, Japan; E-Mail: t-ueda@waseda.jp

**Keywords:** quartz crystal microbalance, high fundamental frequency, wet etching process, rectangle, high Q value

## Abstract

In this study, a miniaturized high fundamental frequency quartz crystal microbalance (QCM) is fabricated for sensor applications using a wet etching technique. The vibration area is reduced in the fabrication of the high frequency QCM with an inverted mesa structure. To reduce the complexity of the side wall profile that results from anisotropic quartz etching, a rectangular vibration area is used instead of the conventional circular structure. QCMs with high Q values exceeding 25,000 at 47 MHz, 27,000 at 60 MHz, 24,000 at 73 MHz and 25,000 at 84 MHz are fabricated on 4 × 4 mm^2^ chips with small vibration areas of 1.2 × 1.4 mm^2^. A PMMA-based flow cell is designed and manufactured to characterize the behavior of the fabricated QCM chip in a liquid. Q values as high as 1,006 at 47 MHz, 904 at 62 MHz, 867 at 71 MHz and 747 at 84 MHz are obtained when one side of the chip is exposed to pure water. These results show that fabricated QCM chips can be used for bio- and chemical sensor applications in liquids.

## 1. Introduction

**Figure 1.**
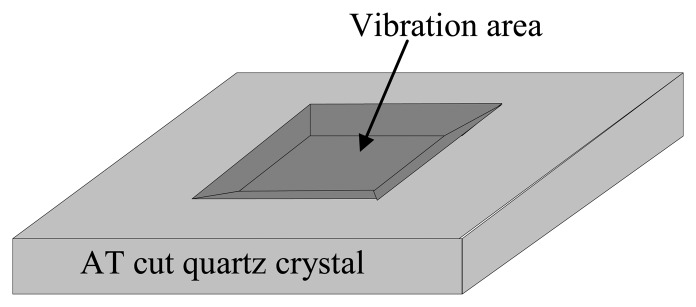
Schematic diagram of an inverted mesa structure for a high frequency QCM.

A quartz crystal microbalance (QCM) is a common mass-sensitive tool that is widely used in chemical and bio-sensor applications. Generally, the QCM resonator functions in a thickness-shear-mode (TSM) vibration mode, and uses an AT-cut quartz crystal for its high temperature-frequency stability. The TSM resonator consists of a thin quartz crystal wafer with two metal excitation electrodes on each side. The QCM measures the frequency shift from the mass change on its surface in accordance with the famous Sauerbrey relation given below [1]:

(1) $$\Delta f=\frac{2f_{0}^{2}\Delta m}{A\sqrt{\rho \mu }}$$

where Δ*f* and Δm are the frequency shift and the mass change, respectively, *f_0_* is the fundamental frequency, A is the electrode area, and ρ and μ are the density and the shear modulus of the quartz crystal, respectively. In theory, increasing the fundamental frequency should dramatically increase the mass-sensitivity, because the frequency shift is proportional to the square of the fundamental frequency for a given mass change per unit area. The fundamental frequency of a TSM resonator depends on its thickness as follows:

(2) $$f_{0}=\frac{\sqrt{\mu /\rho }}{t}$$

where *t* is the quartz crystal thickness. Equations (1) and (2) clearly show that reducing the quartz crystal thickness should enhance the QCM sensitivity. However, in practical manufacturing processes and applications, the minimum thickness is limited by the mechanical strength of the crystals. High frequency TSM resonators are usually fabricated by thinning the central area (*i.e.*, the vibration area) with an inverted-mesa structure using photolithographic and etching techniques. Only the thinned central area vibrates, while the original thickness of the outer ring is retained to supply the necessary mechanical strength for handling and assembling. High fundamental frequency QCMs have been the subject of significant research interest from 1993 to the recent past [2–8]. Using the QCM in a liquid environment produces an unstable vibration, because the liquid flow deflects the vibration quartz membrane. Thus, the thinned vibration area should be manufactured as small as possible, because a small area membrane has a relatively high bending strength. A circular design is usually used for the central thinning area, and the inverted-mesa structure is fabricated using a wet etching process. However, a miniaturized thin vibration area is difficult to manufacture because of the complexity of the etching side wall profile in the wet etching process [9]. The most successful reports to date on miniaturized high frequency QCMs have used a deep reactive ion etching process (DRIE) to produce vertical side walls [9–12]. However, DRIE is an expensive process that requires special etching equipment. The objective of this study is to manufacture a miniaturized high fundamental frequency QCM using a cheap wet etching process. In this research, a simpler side wall profile is obtained by using a rectangular design for the vibration area instead of a circular design (see Figure 1). This research is motivated by the well known result that the etching side wall profile of a quartz crystal is highly dependent on the crystal orientation. Using a resonator as a QCM sensor does not require strict control of the resonating frequency, unlike for resonators used in communication industry. In this case, the frequency shift caused by the mass change is more important than the fundamental frequency; thus we can perform this experimental study without first performing a complicated 3-D simulation. A TSM resonator with fundamental frequency of 622-MHz and a rectangular etching structure has been used in industry [13]. Using a rectangular etching area may enhance the control and predictability of the vibration area. The QCM fundamental frequency (*i.e.*, as determined by the etching area thickness) is controlled by the etching time.

## 2. Experimental Section

### 2.1. Design and Fabrication

**Figure 2.**
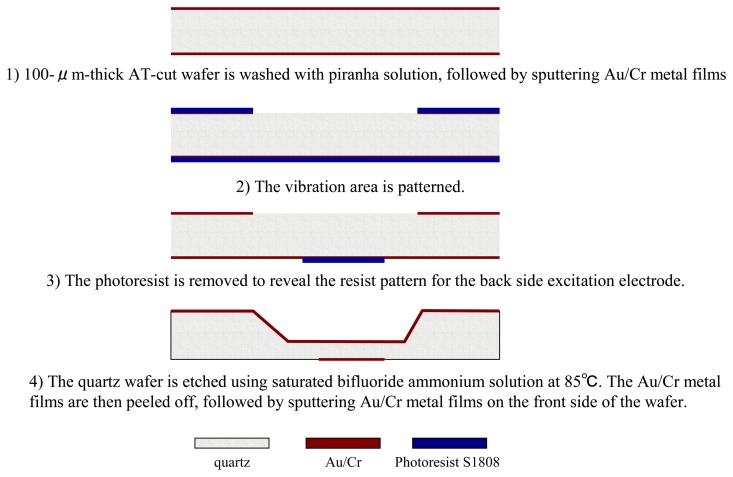
Fabrication process flow of a quartz resonator.

To facilitate mass-production and lower the fabrication costs of QCM chips, the design area of a single QCM chip is set at 4 × 4 mm^2^. The design etching area is 1.2 × 1.4 mm^2^. The initial AT cut wafer is 100 μm thick (to produce the resonance at 16.7 MHz frequency). The design dimensions of the excitation electrode on the front side of the chip range from 200 to 800 μm in diameter with a 100-μm interval. Figure 2 shows the detailed microfabrication process: (1) the quartz wafer is washed using a piranha solution (H_2_SO_4_: H_2_O_2_ = 3:1) at 110 °C, followed by sputtering Au/Cr (100 nm/40 nm) bi-layer metal films onto the wafer to form an etching mask; (2) Au/Cr metal layers are patterned on the back side of the wafer, followed by photoresist removal; (3) a resist pattern is formed on the front side of the wafer to serve as the front side excitation electrode; (4) the quartz wafer is wet-etched using a saturated ammonium bifluoride solution at 85 °C, followed by Au/Cr etching and photoresist removal treatment; (5) Au/Cr is sputtered on the back side of the wafer to form the back side excitation electrode.

### 2.2. Evaluation

**Figure 3.**
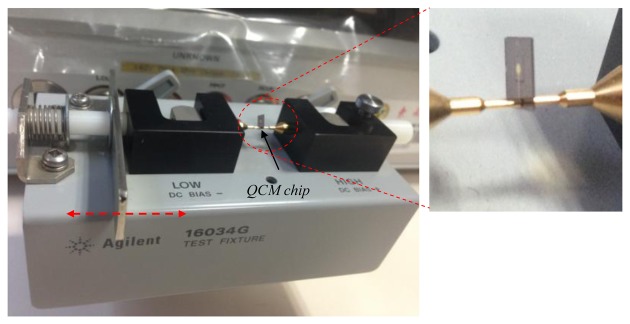
Measurement apparatus with a 16034G test fixture.

**Figure 4.**
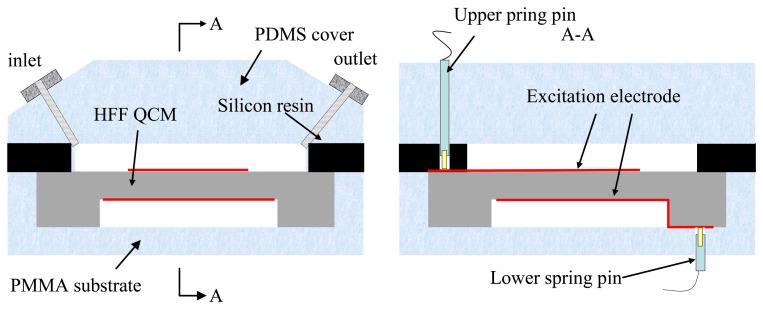
Schematic diagram of the flow cell.

The vibration characteristics (*i.e.*, the Q value, the equivalent circuit parameters, and the condition of the spurious modes near the primary TSM vibration) of the fabricated QCMs are directly evaluated by using an Agilent 4294A impedance analyzer with a 16034G test fixture, as shown in Figure 3. The sweep number is set to 800, and the scanning precision is set at the highest possible value. A flow cell is designed and fabricated to ensure that the chip can be used in practice as a bio- or chemical sensor in a liquid. The custom-designed flow cell consists of a PDMS cover, a thin silicon resin layer, and a PMMA substrate as shown in Figure 4. Liquid can be introduced through inlet and outlet ports in the PDMS cover. The silicon resin layer functions both as a sealing material and a cell chamber. A 100-μm deep cavity is created in the PMMA substrate to stabilize and hold the QCM chip. The excitation electrodes on the two sides of the chip are guided out using two spring-pins, each of which is fixed into a PDMS and PMMA substrate. A simple labview-based program is developed for data acquisition to examine the frequency stability and noise level.

## 3. Results and Discussion

**Figure 5.**
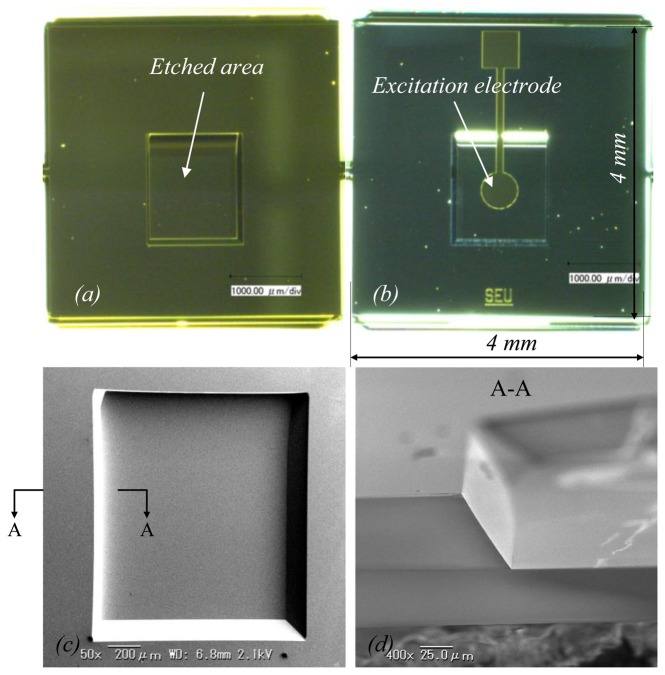
Representative images of a fabricated QCM chip: (**a**) top view; (**b**) back view; (**c**) close-up SEM image of the etched area; and (**d**) cross section of the etched area.

HFF QCMs of 47 MHz, 60 MHz, 73 MHz, and 84 MHz were obtained. Figure 5 shows a fabricated QCM. Figure 5a,b is optical images of the top view and back view of the QCM, respectively. Figure 5c is a close-up image of the etched rectangular area, and Figure 5d is a representative image of the etched side wall profile, which is much simpler than in the circular version.

A high Q value, a low C_0_/C_1_-ratio and a low motional resistance are generally indicative of a high quality resonator [3]. Table 1 shows typical resonance parameters of a 73 MHz series, which are measured using a 4294A impedance analyzer with a 16034G test fixture, as an example.

**Figure 6.**
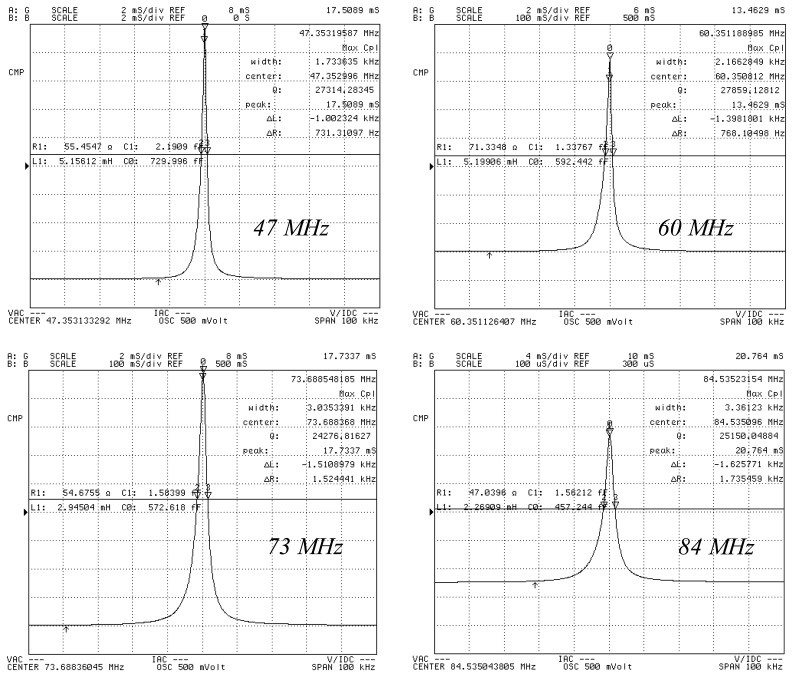
Typical measured vibration characteristics for 47 MHz, 60 MHz, 73 MHz and 84 MHz resonators.

**Figure 7.**
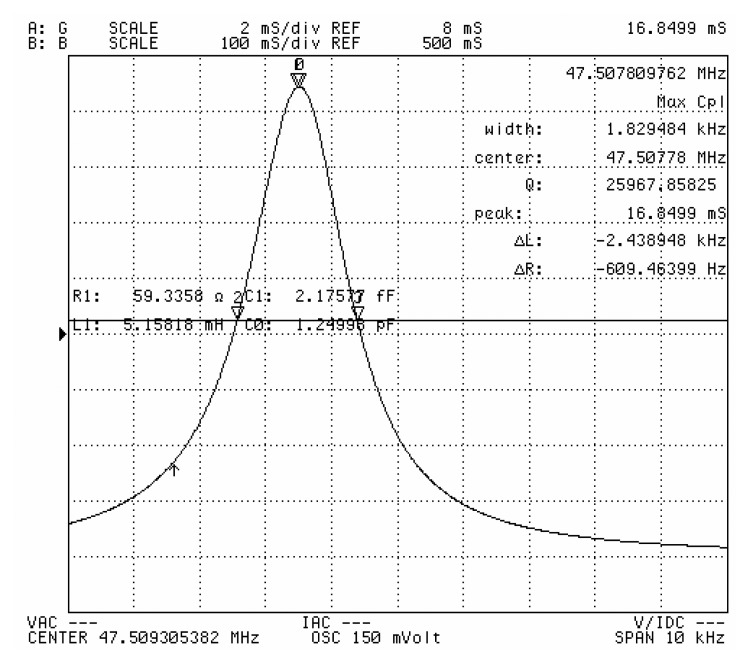
Measured conductance characteristics of a 47 MHz QCM chip in the custom-designed flow cell.

**Figure 8.**
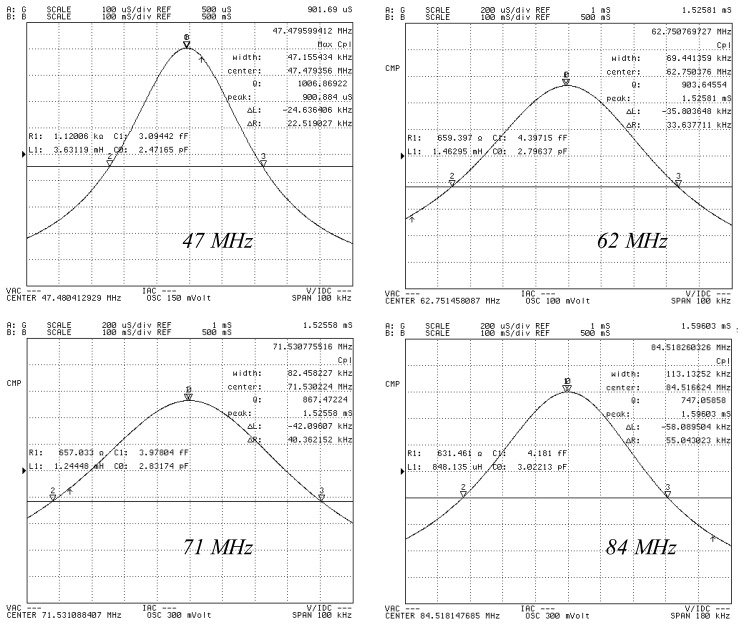
Measured conductance characteristics of 47 MHz, 62 MHz, 71 MHz, and 84 MHz QCM chips in water.

Herein, *f* represents the fundamental frequency, and R_1_, C_1_ and L_1_ represent the motional resistance, the motional capacitance, and motional inductance, respectively. C_0_ represents the shunt capacitance. Table 1 shows that the optimum electrode diameter for a QCM is 500 μm based on the aforementioned evaluation rule. For a given etching area (*i.e.*, the vibration area), the optimal electrode dimension should be a tradeoff between the value from Shockley theory (*i.e.*, the necessary separation between the electrode and the resonator boundary) and Bechmann's number (*i.e.*, the electrode dimension required for energy trapping). For the 47 MHz, 60 MHz, and 84 MHz resonators, the QCM with the highest Q-factor QCM also has an electrode diameter of 500 μm. Figure 6 shows the measured conductance characteristics of the resonators, which are all measured as fabricated. Figure 7 shows the measured conductance characteristics of a 47 MHz QCM chip after it is assembled in the custom-designed flow cell. The Q value of the chip changes from 27,314 to 25,968, following assembly. Thus, the chip is not affected by the flow cell and the electrical leading spring pin. This result shows that the flow cell has been successfully designed and fabricated. Next, all of the four types of QCM chips are assembled in the flow cell, and pure water is flowed through the cell. Figure 8 shows the measured conductance characteristics of 47 MHz, 62 MHz, 71 MHz, and 84 MHz QCM chips in water. The Q values drop to 1,006 at 47 MHz, 904 at 62 MHz, 867 at 71 MHz, and 747 at 84 MHz respectively. The drop in the Q values can be attributed to the high viscosity of water. However, these Q values agree well with the calculated and experimental results in reference [14], and are acceptable for liquid applications according to previous reports [6,14].

All of the Q values of the fabricated high frequency TSM resonators in air exceed 20,000 and are therefore useful for sensor applications. Well known theoretical considerations show that the Q value of the TSM resonator is inversely proportional to the fundamental frequency, for example, a Q value of 25,000 for a 47 MHz resonator corresponds to a Q value of 235,000 for a 5 MHz resonator. In fact, it is very difficult to obtain such a high Q value with a conventional 5 MHz QCM, even for a large diameter of approximately 15 mm. The small size (especially in term of the vibration area size) of the fabricated high frequency resonator translates into a low cost for a single chip, and reduces the sample volume required which is advantageous for sensor applications with expensive or hazardous samples. Furthermore, the small size of a single resonator makes it possible to fabricate a QCM array on one chip for multiple analyses.

**Figure 9.**
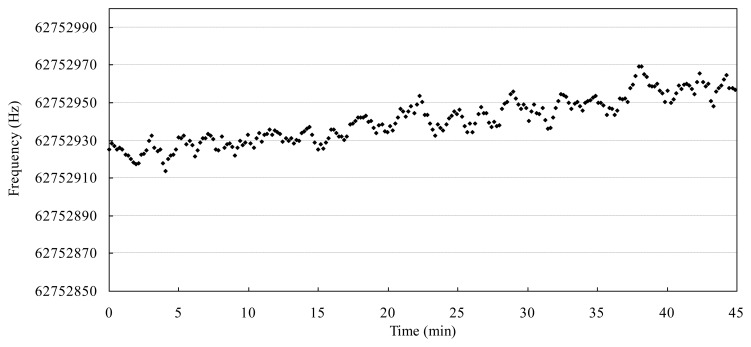
Measured frequency fluctuation of a 62 MHz resonator *via* a 16034G test fixture.

Figure 9 shows the measured frequency fluctuation of a 62 MHz resonator *via* 16,034G test fixture in air and room temperature. The frequency noise (defined as the standard deviation of frequency fluctuation) is measured to be 5 Hz over a period of 600 s (50 points) and the frequency drift (peak to peak) is about 50 Hz over a period of 45 min. The measured noise is higher than the previous reports [6,8,11], although the resonator has the similar mechanical vibration characteristics. Now we are making efforts to reduce the noise level in the following considerations. Firstly, a temperature stabilized chamber is needed to improve the unavoidable temperature noise and drift, which is usually used for precise measurement as above mentioned reports. Secondly, the data processing method should be developed instead of the commercial impedance analysis method [11,15]. Finally, a low bandwidth oscillator circuit is preferred instead of a broadband impedance analyzer [6,16].

## 4. Conclusions

High fundamental frequency QCMs with high Q values up to 25,000 for 47 MHz, 27,000 for 60 MHz, 24,000 for 73 MHz, and 25,000 for 84 MHz resonators were developed in this study. To our knowledge, ours is the first report on fabricating a QCM with an area below 4 × 4 mm^2^, and in particular with a small rectangular etching area of 1.2 × 1.4 mm^2^ using a cheap wet etching process. A PDMS micro-flow cell was designed and fabricated to test the fabricated HFF QCM chips. The flow cell was used to measure Q values of 1,006 at 47 MHz, 904 at 62 MHz, 867 at 71 MHz, and 747 at 84 MHz respectively in water, which are suitable for chemical, and bio-sensors applications in liquids.

## Figures and Tables

**Table 1.** Measured characteristics of 73 MHz QCMs.

**Parameters**	**Electrode Diameter (μm)**						

	**200**	**300**	**400**	**500**	**600**	**700**	**800**
f (MHz)	73.67	73.99	72.54	73.68	71.59	73.09	70.42
Q factor	7,737	13,687	16,692	24,276	18,649	15,157	17,584
R_1_ (Ω)	406	157	77	55	43	40	32
*Υ*(C_0_/C_1_)	1,194	783	563	361	427	473	494
